# Evaluation of insertion quality of a slim perimodiolar electrode array

**DOI:** 10.1007/s00405-023-08212-5

**Published:** 2023-09-29

**Authors:** R. Beck, A. Aschendorff, S. Arndt, T. Hildenbrand, A. K. Rauch, M. C. Ketterer

**Affiliations:** 1 Faculty of Medicine, Department of Otorhinolaryngology, Medical Center – University of Freiburg, University of Freiburg, Freiburg, Germany; 2 Department of Otorhinolaryngology – Head and Neck Surgery, University of Freiburg, Killianstrasse 5, 79106 Freiburg, Germany

**Keywords:** Cochlear implant surgery, Electrode array design, Scalar position, Perimodiolar array

## Abstract

**Objectives:**

The influence of cochlear morphology and electrode array design on scalar position and dislocation rates is of great interest in CI surgery. The aim of this study is to evaluate scalar position and specific points of dislocation in relation to cochlear morphology in patients implanted with a new slim perimodiolar electrode array.

**Materials and methods:**

Patients were implanted using the slim modiolar electrode array (= SMA) (= 532/632 electrode array of Cochlear^™^). Postoperative imaging was performed via cone beam computed tomography (CBCT) and the scans were analyzed regarding cochlear morphology (distances A and B and cochlear height), scalar location of the electrode array, basal insertion depth and apical insertion angle. Furthermore, electrode array design and surgical protocols were evaluated.

**Results:**

81 ears implanted with the SMA were retrospectively included. We evaluated 3 electrode array tip fold over intraoperatively via X-ray imaging and performed revision during the same surgery. The CBCT scans showed 76 initial scala tympani (ST) insertions without dislocation. Two ears showed a dislocated array, one at 77° and the other at 163°. Three arrays were inserted into scala vestibuli (SV) via cochleostomy. These patients showed no signs of obliteration. Cochlear morphology showed no influence on angular insertion depth and scalar position.

**Conclusions:**

The SMA showed a very low rate of scalar dislocations due to its slim electrode array design (2.7%). We could find a learning curve regarding the handling and the risk of dislocation and tip fold over with this electrode array. The rate of intraoperative tip fold over detection via X-ray imaging was 3.7%. Therefore, we highly recommend X-ray imaging and transimpedance matrix measurements within the surgery protocol. Scala vestibuli insertions happened in patients with cochleostomy only. We could identify two specific points of dislocation depending on electrode array design.

## Introduction

Previous research has investigated the relationship between cochlear morphology and the scalar location of electrode arrays [1–6]. Aschendorff et al. [7] and Finley et al. [8] were the first to report improved speech perception outcomes in cochlear implant (CI) patients with scala tympani (ST) location, showing no signs of dislocation and cochlear trauma. Ketterer et al. [3] developed a three-dimensional measurement technique using cone beam computed tomography (CBCT) to assess cochlear distances and height in patients implanted with the Contour Advance electrode array (Cochlear Limited, NSW, Sidney, Australia). Depending on the design, insertion of the electrode array can result in intracochlear malposition, such as the folding of the apical electrode array tip, leading to misstimulation and reduced performance, a condition known as tip fold-over [9–11].

Aschendorff et al. [12] introduced a slim perimodiolar electrode array, the CI 532 (slim modiolar array = SMA = CI 532/CI 632) of Cochlear^™^. Their study included 44 patients, all of whom exhibited complete scala tympani (ST) insertion without any signs of dislocation in radiological assessments. However, they cautioned against over-insertion and tip fold-overs. Ketterer et al. [6] conducted a comprehensive investigation, including the largest cohort to date, analyzing both straight and perimodiolar electrode arrays from MED EL and Cochlear^™^. They identified a distinct angle of dislocation associated with each electrode array design. In addition, they confirmed that the SMA demonstrated no dislocation and appeared to be specifically designed to remain within the initially favored scala, preferably the ST. In contrast to this, other personal reports indicated a significant rate of dislocations and a considerably high number of tip fold-overs of the SMA.

The aim of this study is a radiological and surgical evaluation of results of patients implanted with the SMA regarding scalar position and dislocation behavior of the array itself. Furthermore, we aimed to evaluate the rate of tip fold over in using this perimodiolar electrode array. Therefore, a large study cohort extended from the initial study cohort reported by Aschendorff et al. [12] implanted with the SMA was evaluated retrospectively.

## Methods

Patients implanted with an SMA electrode array were retrospectively included in this study. The patients underwent CI surgery between 2015 and 2020. Preoperatively all patients were evaluated with both high resolution computed tomography and magnetic resonance imaging. Cochlear malformations and patients with otosclerosis or signs of obliteration or ossification were excluded from the study.

We performed postoperative imaging with a CBCT DynaCT-equipped Axium Artis dTA angiography unit (Siemens Co., Erlangen, Germany) and analyzed the images with Impax 6 by Agfa Healthcare via three-dimensional reconstruction. As described by Aschendorff et al. [7, 13] and Ketterer et al. [3, 5, 6], two radiologists and two blinded ENT surgeons evaluated the scalar position of the electrode array and the position of the electrode array dislocation, if any. Cochlear morphology was measured by distance A from the round window through the modiolus and perpendicular distance B as established by Escudé et al. [1]. Cochlear height was determined as described by Ketterer et al. [3]. The cochlear height was only evaluated in one reconstruction, because Ketterer et al. [3] could show that the two different reconstruction possibilities did not differ significantly from each other. Furthermore, the insertion angle was measured between distance A and the apical electrode artefact [3]. We hereby established the angular basal insertion depth from the round window to the first basal electrode artifact (r.w. to b.e.) described before by Holden et al. as most-basal electrode [2].

We performed statistical analysis using Gnu R statistical computation and graphics system (ANOVA, Tukey’s Honest Significant Difference; GNU R, Version 3.0.3, Core Team, Vienna, Austria, http://www.R-project.org). The Ethics Committee of the Albert-Ludwig-University Freiburg approved our study with reference to the Declaration of Helsinki (Washington, 2002) (Number of Ethics Committee approval: 406/19; Amendment: 210,553, 2021) and we registered this study in the German Clinical Trials Register (www.drks.de/DRKS00019807).

## Results

The 81 ears (74 patients) included in this study were all implanted in the Department of Otorhinolaryngology, Head and Neck Surgery and underwent rehabilitation at the Implant Center of the University Hospital Freiburg between 2015 and 2020 (see Table 1). We performed intraoperative X-ray imaging and since established in 2019 transimpedance matrix (TIM) measurements in 32 surgeries to detect tip fold over [9] in all cases and had to revise three patients implanted between 2015 and 2018 due to tip fold over of the electrode array within the same surgery. All tip fold over happened before establishing intraoperative TIM, included in our hospital since May 2019.

**Table 1:** Contribution table of cochlear morphology measurements and electrode array angular apical and basal insertion depth

	Mean	Min	Max	SD
Distance *A* (mm)	9.5	7.8	12.3	0.83
Distance *B* (mm)	6.5	5.1	7.9	0.53
Height (mm)	3.6	2.8	4.5	0.43
Coverage (°)	373	229	480	80
Round window to first basal electrode (= r.w. to b.e.) (°)	16	3	82	12

Distances *A* and *B* were measured recording to Escudé et al. (2006) from the round window to the lateral wall through the modiolus (*A*) and perpendicular to that (*B*)

*Min* minimum, *Max* maximum, *SD* standard deviation

**Fig. 1:**
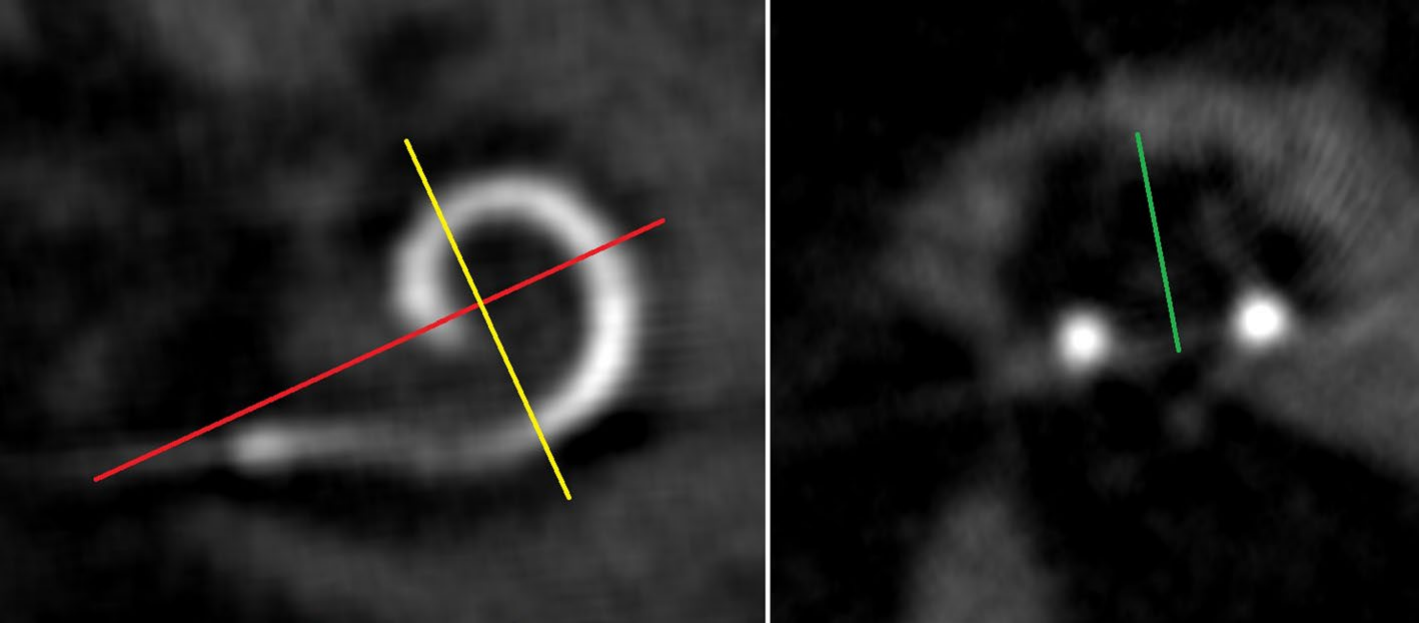
Illustrates the measurements of the cochlear morphology established by Escude et al. [1] (red line: distance A, yellow line: distance b) and Ketterer et al. [3] cochlear height (= green line)

**Fig. 2:**
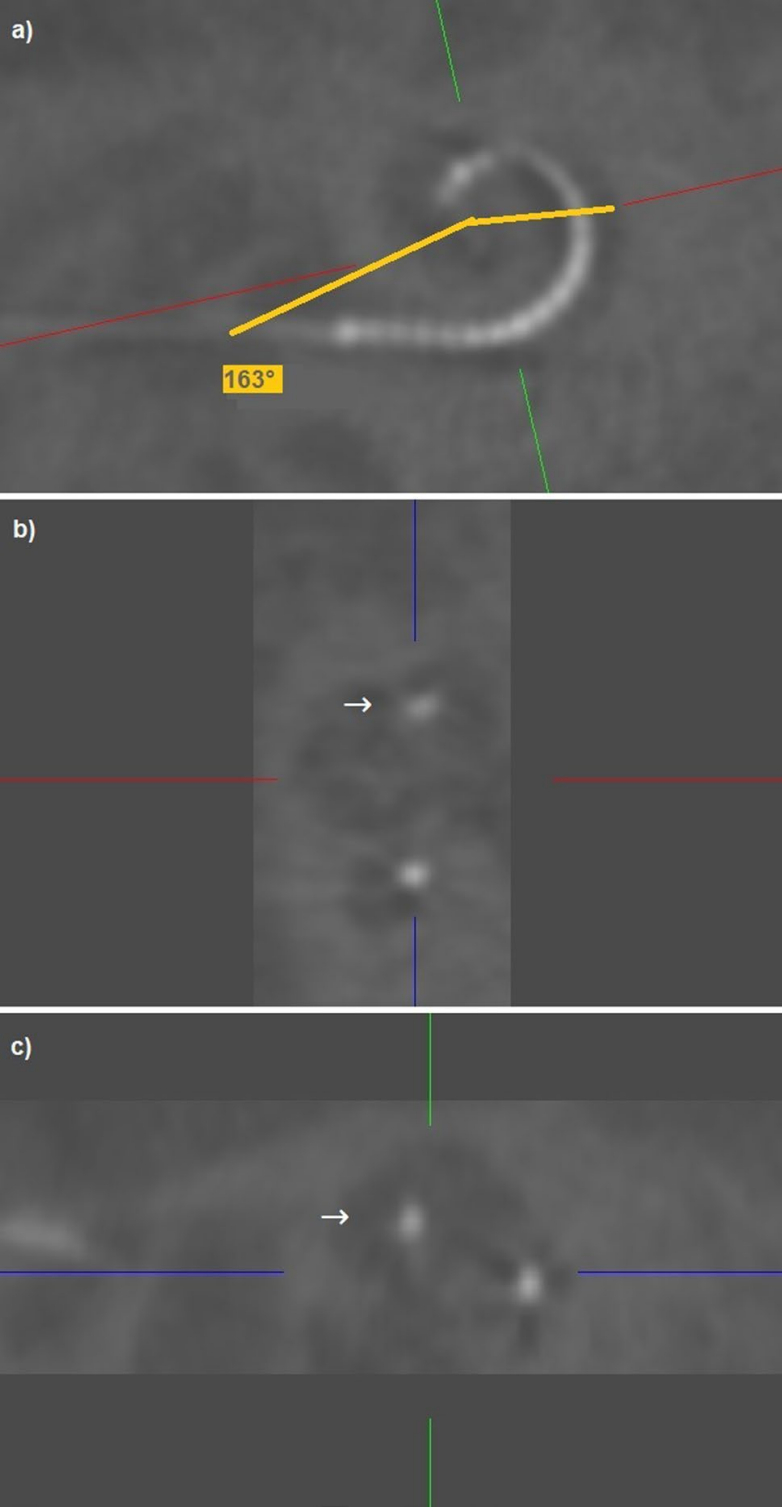
Three-dimensionally reconstructed CBCT: the SMA inserted via round window approach dislocated at 163° (**a**). **b** and **c** show the array initially in ST and clearly in SV after 163° (→)

From the 81 ears, 39 left and 42 right ears were included. Seven patients were implanted with a SMA bilaterally and have been included twice. Evaluation of the cochlear morphology showed a mean distance A of 9.5 ± 0.8 mm and mean distance B of 6.5 ± 0.5 mm (see Table 1). The mean cochlear height [3] was measured as 3.6 ± 0.4 mm. CBCT scalar analysis confirmed ST insertion without dislocation in 76 ears. Two ears showed a dislocated electrode array out of ST. One electrode array (CI532) inserted via cochleostomy in 2017 dislocated at 77°, the other one (CI 532), inserted via round window approach, dislocated at 163° in 2018 (see Figs. 1 and 2). Three ears showed initial SV insertions via cochleostomy (see Table 1).

**Fig. 3:**
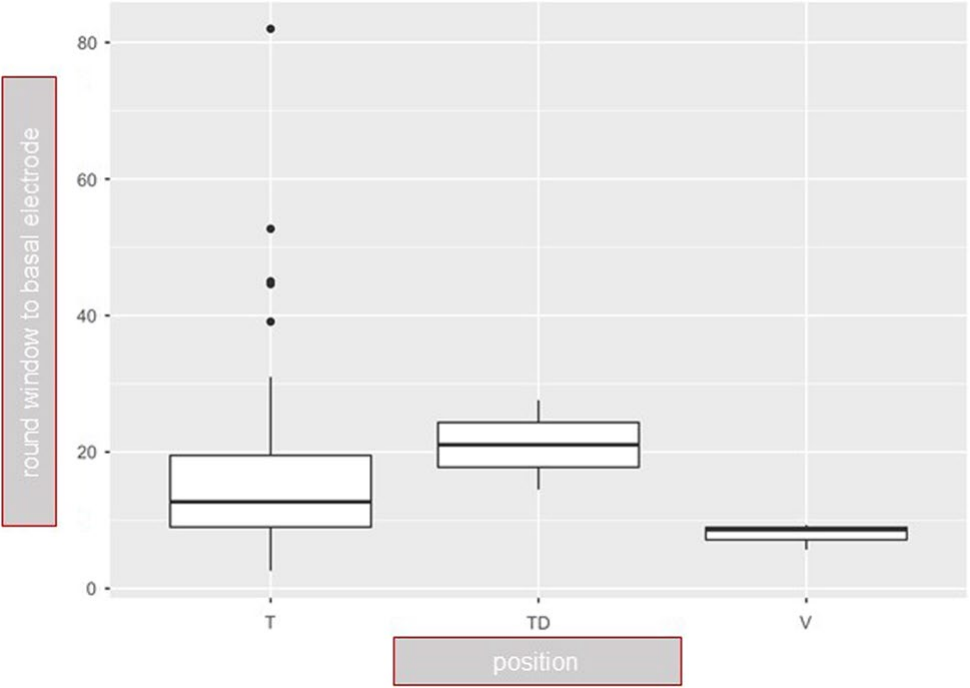
Dislocated electrode arrays initially inserted into scala tympani (TD) showed higher basal insertion depth compared to not dislocated insertions into scala tympani (T) or scala vestibuli (V), but without statistical significance

The mean apical insertion angle was 368° as previously described by Ketterer et al. [3, 5, 6]. The basal electrode insertion depth to the first apical electrode artefact was measured as 16° (see Table 1). Cochlear morphology showed no significant influence on scalar position (distance A versus scalar position (*p* > 0.7). We could find a trend towards a higher risk of dislocation of the electrode array with increasing insertion angle of the first basal electrode, but without statistical significance due to the small number of included patients (Fig. 3). 29 SMA arrays were inserted via cochleostomy, 4 via extended round window approach and 48 via round window approach. The insertion technique comparing cochleostomy and round window approach did not show any significant influence on dislocation rates of the array.

## Discussion

The debate regarding the influence of electrode array design and whether perimodiolar electrode arrays pose a higher risk of scalar dislocation remains unsettled in cochlear implant (CI) surgery. Cochlear morphological measurements from previous studies [1–3, 5, 6, 14] (see Table 1) support these discussions. Aschendorff et al. [7] initially examined scalar dislocation rates in the still-used perimodiolar electrode array Contour Advance (CA = CI512/CI24RECA) from Cochlear^™^ and reported two dislocations in seven initial ST insertions (28.6%). In a subsequent study, Ketterer et al. [3] re-evaluated a larger cohort of patients implanted with the perimodiolar CA and identified 69 dislocations in 319 ST insertions (21.6%). The reduction in translocations can be attributed, in part, to the learning curve of the surgeons [15]. However, a dislocation rate exceeding 20% is still relatively high. To address this, Aschendorff et al. [12] presented the first multicenter results of the SMA, a slimmer perimodiolar electrode array developed to minimize intracochlear trauma. They reported two tip fold-over events in 44 inserted patients and emphasized the need for postoperative radiological evaluation with the SMA. However, no signs of dislocation were found in the patients included in their study [12].

The present study evaluates 78 ears that were implanted with the SMA. Two dislocations (2.7%) were observed, and the position of the dislocation was determined through three-dimensional reconstruction. One electrode array dislocated at 77°, while the other dislocated at 163°. Both dislocations occurred with a CI 532 in 2017 and 2018. In addition, three tip fold-overs were observed within the initial study cohort, all of which occurred prior to 2016. This suggests a learning curve for the SMA, as previously described for the CA [15]. Iso-Mustajarvi et al. [16] inserted the SMA into 20 temporal bones and performed pre- and postoperative CBCT and histological evaluations. They reported 19 round window insertions, one cochleostomy, and 19 insertions without any signs of dislocation. One temporal bone exhibited radiological and histological evidence of a dislocation at approximately 150°. Briggs et al. [17] described three traumatic insertions out of 60 evaluated temporal bones that were inserted with earlier prototypes of the SMA. McJunkin et al. [18] reported three dislocations in 23 inserted ears but did not evaluate the position of the translocations. Iso-Mustajarvi et al. [19] described no scalar dislocations in 17 patients included in their study who were implanted with the SMA. They suggested that the SMA could also be suitable for patients requiring electro-acoustic stimulation, offering full cochlear coverage up to the evaluated insertion angle of 395° in cases of progressive hearing loss following CI. In a retrospective evaluation by Ketterer et al. [6], the largest study cohort to date, 380° cochlear coverage was reported for the SMA. They observed that speech perception outcomes decline as cochlear coverage increases for perimodiolar arrays, and they hypothesized that the initial 180–360° range is particularly important. However, various other studies have reported differing results when examining the impact of cochlear coverage on postoperative speech perception [2, 8, 20–23]. While Canfarotta et al. [21] and Buchman et al. [20] had smaller sample sizes, limiting their statistical power, Baskent and Shannon [24] did not find significant benefits for active electrodes positioned beyond a 360° insertion angle in MED-EL recipients. In a study of 96 patients across 9 arrays, James et al. [4] identified a negative correlation between angular insertion depth and speech perception results.

This present study is an evaluation of 78 ears implanted with the SMA. We found two dislocations (2.7%) and could determine the position of dislocation via three-dimensional reconstruction. One electrode array dislocated at 77°, the other at 163°. Both dislocations happened with a CI 532 in 2017 and 2018.

This study confirms the full coverage of the SMA with 373.2° of insertion angle in a larger study cohort. The point of dislocation was comparable to the results for the perimodiolar CA reported by Ketterer et al. [6] in one dislocation. The other electrode array dislocated very early at 77°. In our opinion, this happened due to a mismatch of the angle of the SMA sheath and the ST. Therefore, we recommend keeping in mind that different dislocation points are possible during insertion due to several contributing factors: e.g., anatomy, surgical experience, mechanical properties—the first 180° due to the designed sheath and the risk of damaging the basilar membrane during insertion. Furthermore, the point at approximately 180° in perimodiolar electrode arrays is more likely to result in dislocations due to their electrode array design, as described by Ketterer et al. [6].

Previous studies hypothesized that the ascending part of the cochlea at 180° is more sensitive for dislocation out of the ST for perimodiolar electrode arrays [6, 24, 25]. Aschendorff et al. [13] described that perimodiolar electrode arrays rotate with an upward direction and touch the outer wall, which leads to perforation of the basilar membrane and traumatic insertion with dislocation out of ST. Nevertheless, histological studies are needed to confirm this theories and radiological findings. TIM is helpful to evaluate tip fold overs within the surgery [9] and to potentially reduce the necessity of intraoperative X-ray imaging. Hans et al. [9] successfully demonstrated that among the 100 patients included in their study, there were no instances of false negatives in detecting tip fold-over using TIM. Consequently, we strongly advocate for the utilization of TIM in the context of SMA, especially given the elevated risk of tip fold-over as reported by Aschendorff et al. [12]. The combination of TIM and electrophysiological measurements has the potential to replace intraoperative X-ray imaging in the future. Presently, our recommendation is to employ both techniques for intraoperative detection of tip fold-over and to undertake necessary revisions within the same surgical procedure.

## Conclusion

In conclusion, this study demonstrates safe and atraumatic insertion of the SMA in a large cohort and shows that the SMA leads to lower dislocation rates compared to other perimodiolar electrode arrays. In contrast to our previous results [6], we report two electrode array dislocations. We could detect surgical learning curves regarding both dislocations and tip fold over, which improved the surgical quality of insertion. Nevertheless, we recommend intraoperative X-ray imaging and/or TIM to identify tip fold overs. Our results indicate that any electrode array has the potential to dislocate, depending on the handling of the array and other factors. Therefore, further developments towards a truly atraumatic electrode array design are necessary.

## Data Availability

The data that support the findings of this study are available on request from the corresponding author.
